# Efficacy of Second-Line Advanced Therapy in Patients with Crohn’s Disease After Failure of a First Anti-TNF: A Descriptive Analysis

**DOI:** 10.3390/jcm15083029

**Published:** 2026-04-16

**Authors:** Corina Meianu, Carmen Monica Preda, Mircea Diculescu, Doina Istratescu, Anca Trifan, Alina Tantau, Ana Maria Singeap, Cristian George Tieranu, Horia Minea, Ana-Maria Buzuleac, Lucian Negreanu, Remus Popescu, Andreea Bota, Tudor Stroie, Letitia Tugui, Andreea-Maria Cazimirovitz, Cosmin Alexandru Ciora

**Affiliations:** 1 UMF “Carol Davila”, Gastroenterology & Hepatology Department, 020021 Bucharest, Romania; corina.meianu@umfcd.ro (C.M.); mihai.diculescu@umfcd.ro (M.D.); cristian.tieranu@umfcd.ro (C.G.T.); lucian.negreanu@umfcd.ro (L.N.); tudor-gheorghe.stroie@umfcd.ro (T.S.); cosmin.ciora@umfcd.ro (C.A.C.); 2 Gastroenterology & Hepatology Department, Clinic Fundeni Institute, 022328 Bucharest, Romania; doina.proca08@gmail.com (D.I.); letitiatugui@gmail.com (L.T.); 3 Department of Gastroenterology, Faculty of Medicine, University of Medicine and Pharmacy “Grigore T. Popa”, 700115 Iasi, Romania; ancatrifan@yahoo.com (A.T.); anamaria.singeap@yahoo.com (A.M.S.); horia.minea@yahoo.com (H.M.); buzuleac.am@gmail.com (A.-M.B.); 4 Institute of Gastroenterology and Hepatology, “St. Spiridon” University Hospital, 700111 Iasi, Romania; 5 Cluj CF University Hospital, UMF “Iuliu Hatieganu” Cluj, 400347 Cluj, Romania; alitantau@gmail.com; 6 Gastroenterology & Hepatology Department, Elias Emergency Hospital, 011461 Bucharest, Romania; andreeadbota@gmail.com (A.B.); andreeacazimirovitz@yahoo.ro (A.-M.C.); 7 Internal Medicine Department, Emergency Universitary Hospital, 050471 Bucharest, Romania; remus-florin.popescu@drd.umfcd.ro

**Keywords:** Crohn’s disease, treatment sequencing, biologic therapy, second-line therapy, real-world data, infliximab, adalimumab, ustekinumab, vedolizumab

## Abstract

**Introduction:** Sequencing therapy for Crohn’s disease (CD) is currently being intensively discussed due to the development of novel drugs and lack of standardized criteria for drug positioning in first- and further-line treatment. The aim of this study was to compare the efficacy of a second-line advanced therapy in Romanian patients with CD who have failed an anti-TNF agent. **Methods:** We performed a multicenter retrospective study that included adult patients with CD who had secondary loss of response after an initial response with an anti-TNF drug. The main outcome was clinical remission at 12 weeks of second-line treatment (CDAI < 150). The secondary outcomes included clinical response (decrease in CDAI ≥ 70 points), persistence of therapy at 1 year and rates of adverse events. **Results:** From 2008 to 2024, 216 patients were either switched to another anti-TNF or swapped to another therapeutic class, due to the failure of a first anti-TNF drug. Secondary lines of treatment included infliximab (IFX), adalimumab (ADA), vedolizumab (VDZ), ustekinumab (UST). The highest rate of clinical remission (81%) was obtained with the sequence ADA-IFX in 26/32 (81%) patients and ADA-UST in 62/82 (76%) patients, followed by IFX-UST in 22/33 (67%) and IFX-ADA 34/51 (67%). Persistence in therapy at 1 year was better for the sequence ADA-UST (73%) and IFX-UST (67%) and ADA-IFX (63%) compared to IFX-ADA (59%) and IFX-VDZ (44%) (*p* < 0.001). **Conclusions:** There were significant baseline differences between the treatment groups, so this study represents an unadjusted comparison between the results obtained with different biologics in second-line treatment for Crohn’s disease. In patients with CD who have failed a first anti-TNF, the highest rate of clinical remission at 12 weeks was obtained with second-line IFX and UST whilst vedolizumab showed lower efficacy. UST demonstrated the most favorable long-term treatment persistence at 1 year.

## 1. Introduction

Crohn’s disease (CD) is an inflammatory bowel disease that causes relapsing inflammation in the gastrointestinal tract that can lead to irreversible bowel damage and an increase in disability. The physiopathology of the disease is explained by an abnormal immune response to a complex interplay of factors, including genetic susceptibility, environmental triggers, and an alteration in the gut microbiota [1,2]. Incidence of CD is increasing worldwide and all age groups can be affected, from children to the elderly [3]. At the moment, there is no treatment that can permanently cure the disease, which leads to high clinical, social, economic and psychological burden [4]. Since 1990, starting with the approval in Europe of infliximab as the first biologic agent for the treatment of CD and followed by adalimumab and golimumab, the use of anti-TNF has significantly improved CD outcomes [5,6,7,8,9]. Despite the initial efficacy, a significant proportion of patients experience secondary loss of response to anti-TNF drugs or develop adverse reactions or intolerance, necessitating a change in therapy [10]. In the last 10 years, significant advances have been made in the field of CD with the development of new molecules that target different immunological pathways, with promising results in achieving disease control [11]. Alongside the expanding therapeutic landscape and improved insight into the natural history of CD, there has been a paradigm shift toward tighter disease control. As a result, treatment goals have progressed beyond clinical remission, aiming for deeper endpoints, including endoscopic healing and even transmural remission [12,13]. Emerging data demonstrates the decreased efficacy of advanced therapy when used in bio-experienced patients when compared with bio-naïve patients, highlighting that the choice of second-line therapy could be a key determinant of long-term disease control [14,15].

For patients with CD following anti-TNF failure, there is still insufficient evidence to determine the optimal choice of second-line therapy [11]. This topic remains an area of active research. One of the main controversies is whether patients who experience secondary loss of response to an anti-TNF agent should be switched to a different agent within the same class or should transition to a drug with a different mechanism of action [16]. Currently, there are no definitive position statements or guideline-endorsed algorithms for the selection of a second-line therapy after anti-TNF failure. The existing literature data come from network analysis, metanalysis and retrospective observational studies based on real-world clinical cohorts [17,18,19]. More extensive real-world data and larger cohort studies are needed to better inform second-line therapeutic strategies.

Our study aimed to assess the comparative efficacy of second-line biologic therapies in a multicenter cohort of Romanian patients with CD who experienced secondary loss of response to a first anti-TNF agent.

## 2. Material and Methods

### 2.1. Participants and Eligibility

We conducted a retrospective study, which enrolled all patients diagnosed with and treated for Crohn’s disease between January 2008–December 2024, registered in the Romanian inflammatory bowel disease database (IBD Prospect), from six Gastroenterology departments across Romania: academic centers from Bucharest, Iasi and Cluj. The enrolled patients met the inclusion criteria as outlined below: 547 patients with Crohn’s disease on biologic therapy were screened for the study, and 295 (55%) were excluded because they remained on first-line biologic. Of the 252 people who required a switch to second-line treatment, 36 patients had Ustekinumab as their first line of treatment, so they were also excluded from the study.

The study included patients diagnosed with CD based on standard diagnostic criteria, as determined by the physicians who had been receiving anti-TNF agents—IFX (infliximab) or ADA (adalimumab)—for at least 3 months, had an initial response to therapy as defined by a decrease in the Crohn’s Disease Activity Index (CDAI) with at least 70 points, had a secondary loss of response defined by clinically active disease (CDAI > 150) and had received at least 12 weeks of therapy with a second-line biologic agent. All patients included in this study had CDAI calculated every 3 months under advanced biological treatment. In the case that CDAI was above 150 points, CRP and fecal calprotectin were determined. In case of elevated CRP and calprotectin above 150 mcg/g, patients underwent colonoscopy and/or CT imaging or intestinal ultrasound to highlight disease activity and confirm secondary loss of response to treatment.

In the case of secondary loss of response, according to the national protocol for treatment with biological agents in IBD, therapeutic drug monitoring is performed: in the case in which the serum level of ADA or IFX is suboptimal, the dose is optimized; if anti-ADA or anti-IFX antibodies are present, immunosuppressive treatment is added. If the serum level is optimal, a switch within the class or swap is made (the treatment is changed to a biological agent from another therapeutic class: UST or VDZ).

The choice of second-line therapy after anti-TNF failure was made at the discretion of the treating physician, based on clinical judgment and local practice. No randomization was possible in our study, as it was a retrospective cohort study.

IBD phenotype; history of surgical interventions; treatment initiation and discontinuation dates; diagnostic delay (defined as the time from first symptoms to diagnosis); the interval from diagnosis to initiation of first biologic therapy; and levels of fecal calprotectin, hemoglobin, and C- reactive protein at the time of biologic switch and periodically during disease monitoring were all systematically recorded.

All patients provided written informed consent.

The study was approved by the Ethics Committee of the Fundeni Clinical Institute.

### 2.2. Statistical Analysis

The main outcome was clinical remission at 12 weeks of second-line treatment, defined as a Crohn’s Disease Activity Index (CDAI) < 150. Secondary outcomes included clinical response at 12 weeks, defined as a decrease in CDAI ≥ 70 points, treatment persistence at 1 year, and rates of reported adverse events. Patients who failed to achieve either remission or response were classified as primary non-responders.

Statistical analyses were performed using SPSS software (version 20.0, IBM Corporation, Armonk, NY, USA). The normality of continuous variables was assessed using the Kolmogorov–Smirnov test. Normally distributed variables were expressed as mean ± standard deviation, whereas non-normally distributed variables were summarized as the median and range (minimum–maximum).

For comparisons between the treatment sequence groups, one-way analysis of variance (ANOVA) was used for normally distributed variables, while the Kruskal–Wallis test was applied for variables with non-normal distribution. Categorical variables were expressed as frequencies and percentages and compared using the chi-square test or Fisher’s exact test, as appropriate.

All statistical tests were two-sided, and a *p*-value < 0.05 was considered to be statistically significant. Given the exploratory nature of the analyses and the presence of multiple comparisons across treatment sequences, no formal adjustment for multiple testing was applied, and the reported *p*-values should therefore be interpreted with caution.

## 3. Results

### 3.1. Biologic Therapy Utilization and Baseline Cohort Characteristics

**Figure 1:**
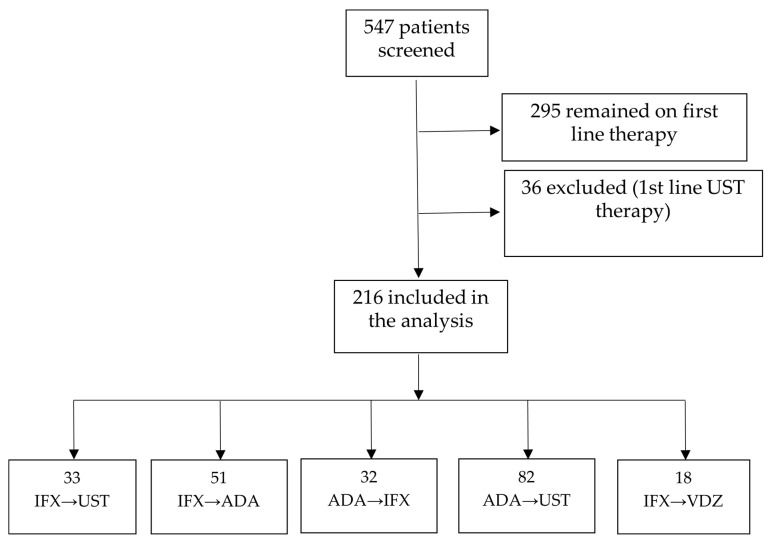
Study flow-chart.

Figure 1 is the flowchart of the study.

Following the data screening and application of the eligibility criteria, a total of 216 patients who had failed first-line anti-TNF therapy were included in the final analysis. The follow-up duration had a median of 48 months (minimum 24 months and maximum 180 months). The baseline characteristics of the overall cohort are summarized in Table 1.

Second-line therapies were represented by a second anti-TNF (IFX or ADA), UST (ustekinumab) and VDZ (vedolizumab). The most common second-line treatment sequence was ADA, followed by UST in 82 (32.5%) of patients. This was followed in frequency by the IFX to ADA sequence in 51 (20.2%) patients, IFX to UST in 33 (13.2%) patients, ADA to IFX in 32 (12.7%) patients and IFX to VDZ in 18 (7.1%) patients. The highest CDAI scores at second-line therapy initiation were observed in the group of patients who failed IFX as a first-line treatment and were treated with VDZ as a second-line treatment. The highest levels of fecal calprotectin were observed in the IFX failure group and received as second-line therapy UST, ADA or VDZ. Perianal disease was observed most frequently among patients who experienced first-line failure with IFX and were subsequently treated with ADA as second-line therapy, comprising 18/51 (35.4%) patients. The stricturing phenotype was predominantly found among patients who failed ADA and were most frequently switched to UST 34/82 (41.5%) and IFX 24/32 (75%) patients, respectively. Significant differences were recorded in the proportion of patients who received immunosuppressive treatment at the initiation of second-line biological treatment, with 71.9% of people who received Infliximab in second-line treatment being treated with Azathioprine combination therapy, followed by those who received Adalimumab (31.4%). Regarding steroids at the baseline, most individuals received steroids at the initiation of the second biologic treatment, with the proportions varying between 56.3 and 88.9%. Patients’ characteristics across different second-line biologic treatment strategies are shown in Table 2.

### 3.2. Efficacy of Second-Line Therapy After Anti-TNF Failure

Given the significant baseline differences between treatment sequence groups (Table 2), including variations in age, smoking status, disease phenotype, prior surgery, and inflammatory markers, the analyses presented in this study represent unadjusted comparisons. Therefore, the results should be interpreted as descriptive associations between treatment sequences and outcomes, rather than as evidence of causal effects or the superiority of one sequence over another.

Overall, patients reported a complete clinical response rate of 70.7% at 12 weeks and a partial response rate of 11.8%, while the non-response rate was 16.8%.

**Figure 2:**
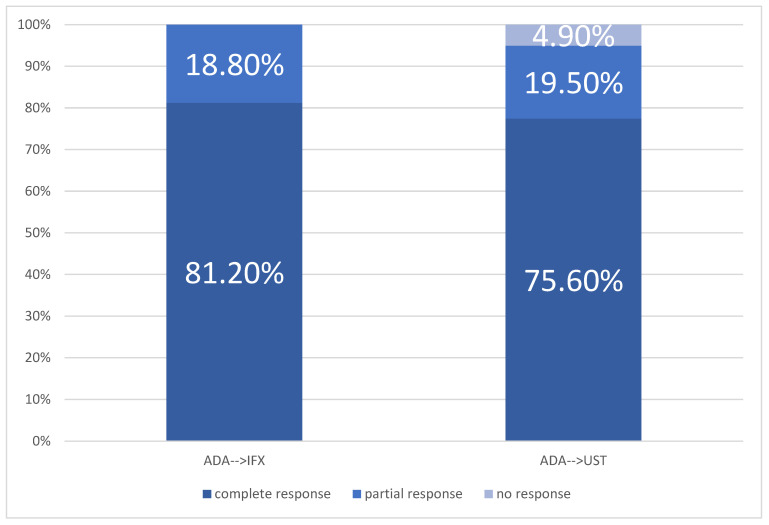
Rate of response in patients treated with ADA as 1st line sequence.

Figure 2 shows the comparative results obtained with second-line biological therapy when patients were treated in the first line with Adalimumab.

Clinical remission rates at 12 weeks varied across treatment sequences. In the ADA→IFX sequence, 81.2% of patients (26/32) achieved clinical remission, while 75.6% (62/82) of patients treated with ADA→UST achieved remission.

**Figure 3:**
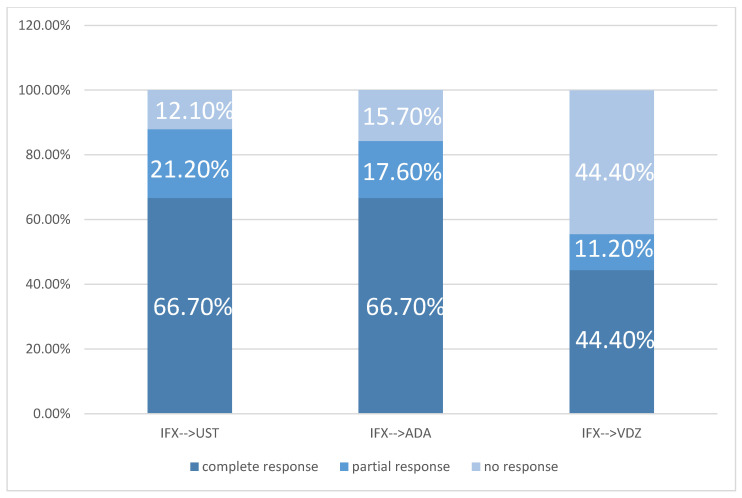
Rate of response in IFX as 1st line sequence.

Clinical response rates at 12 weeks when Infliximab was used in the first line are represented in Figure 3. IFX→UST and IFX→ADA groups both had a 67% remission rate (n = 22/33 and 34/51), respectively (*p < 0.001* for comparison with people who received first-line treatment with ADA).

The highest partial clinical response rate at 12 weeks was observed in the IFX→UST group (21%, 7/33), followed by ADA→IFX (19%, 6/32), IFX→ADA (18%, 9/51) and ADA→UST (17%, 14/82), *p < 0.001*.

Primary non-response to second-line therapy was observed in the IFX-UST group (21%, 7/33), followed by ADA-UST (17%, 14/82), IFX-ADA (18%, 9/51), and IFX-VDZ (17% 3/18), *p < 0.001*. No cases of primary non-response were recorded in the ADA-IFX group.

**Figure 4:**
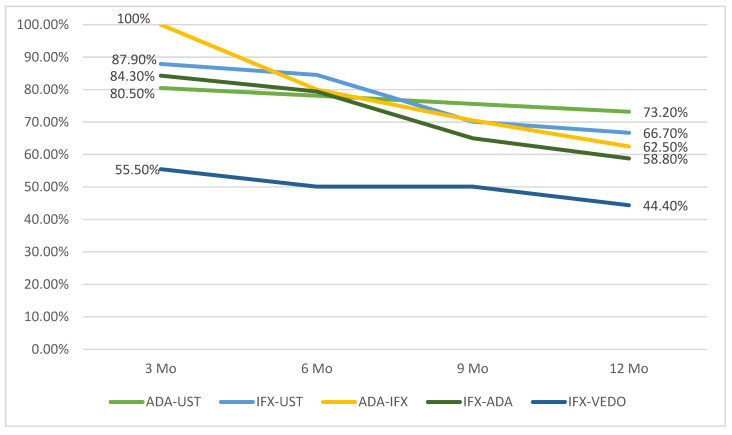
Persistence of treatment over one year with second-line biological therapy (on different therapeutic sequences).

In Figure 4, we represented the persistence of treatment over a year with second-line biological therapy (on different therapeutic sequences). Persistence was defined as the proportion of patients remaining on treatment at predefined time points (3, 6, 9, and 12 months).

For the calculation of persistence at each time point, only patients with available follow-up at that specific time point were included in the denominator. Patients who had a shorter follow-up duration than the evaluated time point were not considered treatment failures, but were excluded from that specific time-point analysis.

At 12 months, 73% of patients (60/82) in the ADA→UST sequence remained on treatment. The persistence rates were 67% (22/33) for IFX→UST, 62.5% (20/32) for ADA→IFX, 59% (30/51) for IFX→ADA, and 44% (8/18) for IFX→VDZ. These differences were statistically significant (*p* < 0.001). A total of 43 patients out of 216 (20%) discontinued second-line biological treatment: most due to loss of response (38, that is 17.5%), and only a minority (5) due to adverse events. Four adverse events were recorded in the group ADA→IFX, one severe allergic reaction and three severe bacterial infections. In the IFX→ADA group, a severe bacterial infection (pulmonary tuberculosis) was the reason for the discontinuation of second-line biological treatment.

Because treatment groups differed substantially in the baseline clinical characteristics, these findings should be interpreted as descriptive comparisons, rather than adjusted estimates of treatment effectiveness.

Of the total 216 patients included in the statistical analysis, only 65 (30%) had a control colonoscopy at 12 months of second-line biological treatment, and because this number of patients was very small, we were unable to perform a statistical analysis regarding the endoscopic response to treatment.

**Figure 5:**
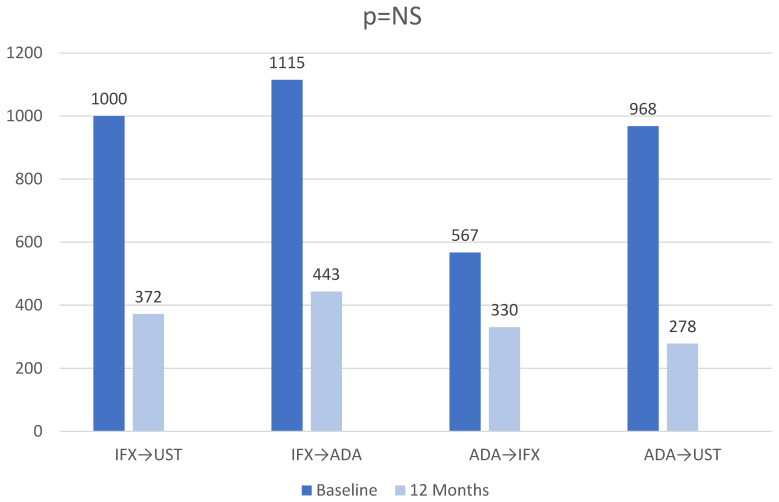
Evolution of fecal calprotectin levels 12 months after initiation of 2nd-line biological medication.

Figure 5 describes the evolution of fecal calprotectin levels at 12 months after the initiation of second-line biological medication. The median calprotectin levels evolved favorably in all therapeutic sequences, with the largest decrease of 690 mcg/g being recorded among the ADA→UST patient group, but without achieving statistical significance between the four treatment sequences (*p*-value is calculated for the difference between fecal calprotectin levels at 12 months, compared between the four therapeutic sequences).

## 4. Discussion

Anti-TNF agents were the first biologic therapies introduced for the treatment of moderate-to-severe CD, and despite the emergence of multiple other monoclonal antibodies and therapeutic molecules, they continued to be widely used as a first-line biologic therapy in CD [11,20,21]. Real-life data from Romania shows that Infliximab and Adalimumab have similar efficacy and safety profiles in the treatment of Crohn’s disease [22,23].

However, long-term data have consistently shown high rates of loss of response after an initial favorable response (secondary loss of response) in more than 50% of patients over exposure to anti-TNF agents [24,25,26,27]. Our study screened 547 persons in total, but 55% were excluded, because they persisted with Infliximab or Adalimumab, a proportion similar to the data in the literature; we assessed the comparative efficacy of second-line biologic treatments in a multicenter cohort of 216 Romanian patients with CD who experienced secondary loss of response to an initial anti- TNF therapy and we evaluated the 1-year persistence to second-line treatment as a measure of long-term therapeutic efficacy. It was not possible to randomize patients to the different therapeutic sequences used; in this retrospective cohort study, physician-driven allocation was performed. Patients in this cohort were treated with both the original molecules and biosimilars, given that the existing studies demonstrate that they have comparable efficacy and safety [28,29]. When comparing clinical outcomes across second-line biologic sequences, we found that the ADA-IFX sequence demonstrated the highest rate of clinical remission (81%) at 12 weeks. The guideline recommendations suggest that in the case of primary non-response to a first anti-TNF agents, switching to a biologic treatment with a different mechanism of action is advised. Conversely, in cases of secondary loss of response, transitioning to a second anti-TNF agent within the same class may be considered [20]. However, the real-world evidence and randomized clinical trial data are conflicting. Kapizioni C. et al. demonstrated that VDZ and UST both offer similar superior efficacy as second-line therapy in CD following anti-TNF failure, rather than a second anti-TNF [15]. Also, results from Attauabi M. et al. network metanalysis (NMA) concluded that in bio-exposed CD patients upadacitinib and risankizumab ranked highest in inducing clinical response (including adalimumab exposed patients); however, infliximab was not investigated in this group [18]. In a NMA, Singh S. et al. suggests ADA (after secondary loss of response to IFX) or Risankizumab as preferred second-line therapy in CD [30]. Most of these studies did not differentiate between secondary loss of response due to immunogenicity, where switching to another anti-TNF might still be effective, and loss of response occurring despite adequate drug levels, which would suggest a loss of response due to the ineffective mechanism of action. Furthermore, anti TNF therapeutic drug monitoring was not performed in most studies at the point of treatment failure. Consistent with the limitations observed in previous research, our study did not include therapeutic drug monitoring at the time of secondary loss of response. Furthermore, in both ulcerative colitis and CD, IFX and ADA efficacy does not appear to be equivalent, even at therapeutic drug levels. In perianal fistulizing CD, guideline recommendations suggest anti-TNF therapy, with IFX being preferred over ADA [11]. While comparative data in CD remain limited, stronger evidence exists in UC, where IFX has demonstrated superior efficacy over ADA [30]. In our cohort, sequences including second-line IFX or UST were associated with higher crude rates of clinical remission after adalimumab failure. This finding contributes additional evidence in positioning the efficacy of anti-TNF agents in CD and suggests that IFX may be an effective option for patients who fail first-line ADA therapy. Our findings are consistent with the previously published data that position UST as a high-potency molecule in the second line after anti-TNF therapy [16,31,32,33]. The lowest rates of clinical remission were observed in the IFX-VDZ group in 44% (8/18). Along with the existing literature data, our findings suggest that VDZ may be a better option as a first-line therapy in CD [34,35,36]. However, it is important to take into consideration the small sample size included in our study and that the majority of patients—55.6% (10/18)—had perianal CD. Considering the CD phenotype, the highest proportion of perianal fistulizing CD was seen in the IFX-ADA group—35% (18/52) patients—in which the primary endpoint of clinical remission after induction was achieved at a significant rate of 67%. The highest proportion of stricturing CD was seen in the ADA-IFX group, followed by the ADA-UST group: 75% (24/32) patients and 41.5% (34/82) patients, respectively. Both groups achieved clinical remission at 12 weeks with significant rates of 81% and 76%. The data supports the benefit of these sequences of biologic treatments after anti-TNF failure in this subgroup of patients.

The highest persistence rate at 1 year was observed in the ADA-UST group, where 73% of patients (n = 60/82) remained on therapy, followed by IFX-UST with a persistence rate of 67% (n = 22/33). The results are consistent with the favorable long-term persistence profile of UST reported in the literature [36,37].

The ADA-IFX group has also shown significantly high rates of treatment persistence at 1 year: 62.5% (20/32 patients). This significantly better persistence of the biological treatment when ADA was prescribed in the first line is also described in a recently published study by Louis E. et al., who used a semi-Markov sequential model in order to identify the optimal position of ADA and VDZ in the treatment sequence in Crohn’s disease [33].

Our study has several limitations. Firstly, the relatively small study cohort may limit the generalizability of our findings. Secondly, the retrospective design is inherently associated with potential selection bias, missing data, and unmeasured confounders. The most important limitation is that there are statistically significant differences between the patients included in the five therapeutic sequences (IFX→UST, IFX→ADA, ADA→IFX, ADA→UST, IFX→VDZ) in terms of age at onset, disease location, evolutionary pattern, proportion of smokers, proportion of people with perianal fistulas and those who required surgical interventions for resection of the small intestine or colon. These variables are known predictors of treatment response and persistence in Crohn’s disease. Because the analyses presented are unadjusted, the observed differences in remission and persistence across treatment sequences should be interpreted as associations, rather than causal treatment effects. Future studies using larger prospective cohorts that systematically incorporate TDM and immunogenicity data, including newer agents (risankizumab, upadacitinib, etc.) for CD therapy and multivariable or propensity-score-adjusted analyses are needed to better evaluate the comparative effectiveness of different biologic sequencing strategies. Another limitation of this study is the lack of data on endoscopic remission at 12 months in the different therapeutic sequences used, since only 30% of patients had a control colonoscopy after initiating second-line biological therapy; however, the fecal calprotectin values recorded a significant decrease in all treatment arms, which was consistent with persistence in treatment.

While acknowledging these limitations, it is important to highlight the study’s main strengths. We performed a multicenter analysis that provides real-world evidence in a field in which data on second-line biologic sequencing in CD remain limited. Moreover, we included long-term assessment of treatment persistence at 1 year, adding clinically relevant information to guide therapeutic decision making.

However, the therapeutic prospects in inflammatory bowel diseases are expanding from year to year with the emergence of new biological agents and small molecules, and bacteria-based platforms and protein-based nanoparticle drug delivery systems (PNP-DDSs) are now in the preclinical testing phase [38,39].

## 5. Conclusions

In our multicenter real-world cohort of Romanian patients with CD experiencing secondary loss of response to a first anti-TNF, there were significant baseline differences between the treatment groups, so this study represents an unadjusted comparison between the results obtained with different biologics in second-line treatment in Crohn’s disease. Our descriptive analysis suggests that for patients failing ADA with secondary loss of response, IFX and UST appear to be reasonable second-line options, particularly in those with stricturing or perianal disease.

For patients failing IFX, UST may provide better long-term persistence than VDZ in this setting, although the baseline differences limit firm conclusions.

These findings provide additional evidence to guide second-line biologic sequencing in CD.

## Figures and Tables

**Table 1:** Baseline characteristics of the overall cohort.

Number of Patients	216 (100%)
Sex	Males—129 (59.7%) Females—87 (40.3%)
Age (median, min–max)	33 (11–69)
Smokers	Active Smokers—86 (39.7%) Ex-smokers—17 (7.9%) Non-smokers—113 (52.4%)
BMI (median, min–max) (kg/m^2^)	23.38 (10.34–40.40)
Location-ileal	63 (29.2%)
Location-ileo-colonic	63 (29.2%)
Location-colonic	90 (41.6%)
Non-stricturing, non-fistulizing	114 (52.8%)
Stricturing	66 (30.4)
Perianal disease	35 (16.4%)
Surgical intervention	81 (31.9%)
CDAI (median, min–max)	245 (150–557)
Calprotectin (median, min–max) (µg/g)	650 (3.30–7762)
Hb at initiation (median, min–max) (g/dL)	12.7 (5.4–17)
CRP at initiation (median, min–max) (mg/dL)	11.2 (0.15–270)
Latency from symptoms to diagnosis (months) (median, min–max)	7 (1–288)
Interval diagnosis–biologic initiation (months) (median, min–max)	8 (1–444)

BMI: Body mass index, CDAI: Crohn’s Disease Activity Index, Hb: hemoglobin, and CRP: C-reactive protein.

**Table 2:** Patient characteristics by type of second-line biologic therapy.

	IFX→ UST	IFX→ ADA	ADA→ IFX	ADA→ UST	IFX→ VDZ	*p*-Value
Patient No (%)	33 (13.1%)	51 (20.2%)	32 (12.7%)	82 (32.5%)	18 (7.1%)	
Age (median, minim, maxim	25 (17–42)	28 (11–35)	29 (28–39)	34 (18–69)	37 (27–50)	<0.001
Smoker No (%)	4 (12.1%)	28 (54.9%)	10 (31.3%)	38 (46, 3%)	12(66.7%)	<0.001
BMI (median, minim, maxim)	23 (18–28.4)	21 (19–30)	24 (22–26)	21 (18–40.4)	24 (21–27)	0.005
L1 *: ileal	L1: 5 (15.2%)	L1: 15 (29.4%)	L1: 18 (56.2%)	L1: 26 (31.7%)	L1—0 (0%)	<0.001
L2 *: colonic	L2: 8 (24.2%)	L2: 22 (43.1%)	L2: 2 (6.2%)	L2:20 (24.4%)	L2—10 (55.6%)	
L3 *: ileo-colonic	L3: 20 (60.6%)	L3: 14 (27.5%)	L3: 12 (37.6%)	L3: 36 (43.9%)	L3—8 (44.4%)	
Non-stricturing non penetrating: No (%)	19 (57.6%)	23 (45.1%)	6 (18.8%)	40 (48.8%)	14 (77.8%)	<0.001
Stricturing: No (%)	12 (36.3%)	20 (39.2%)	24 (75%)	34 (41.5%)	2 (11.1%)	<0.001
Perianal (%)	6 (18.2%)	18 (35.3%)	12 (37.5%)	10 (12.2%)	10 (55.6%)	<0.001
Surgery (%)	18 (54.5%)	24 (47.1%)	20 (62.5%)	34 (41.5%)	2 (11.1%)	<0.001
CDAI (median, minim, maxim)	260 (150–557)	224 (156–390)	216.5 (150–370)	260 (150–520)	350 (162–432)	<0.001
Calprotectin (median, minim, maxim) (µg/g)	1000 (236–2950)	1115.96 (3.3–2500)	567 (345–1100)	968 (123–3241)	1050 (111–2325)	<0.001
Hb at initiation (median, minim, maxim) (g/dL)	11.8 (8.60–16.10)	13.1 (10–15.6)	12 (10–15.8)	12.9 (6.4–16.6)	11.4 (9–13.2)	0.010
CRP at initiation (median, minim, maxim) (mg/dL)	8 (2–37)	12 (1.11–116)	22.9 (7–200)	11.1 (0.15–120)	45 (12–101)	<0.001
Latency from symptoms to diagnosis (months) (median, minim, maxim)	8 (1–15)	6 (1–120)	12 (4–240)	5 (1–72)	8 (2–36)	<0.001
Interval from diagnosis to biologic initiation (months) (median, minim, maxim)	8 (1–276)	10 (1–444)	11 (1–144)	8.5 (1–108)	84 (3–120)	0.016
Concomitant immunosuppressants (Azathioprine) at initiation of 2nd-line biologic. No (%)	0 (0%)	16 (31.4%)	23 (71.9%)	0 (0%)	0 (0%)	<0.001
Proportion of patients who received steroids at initiation of 2nd-line biologic. No (%)	25 (75.8%)	41 (80.4%)	18 (56.3%)	58 (70.7%)	16 (88.9%)	0.02

* According to the Montreal classification. ADA: adalimumab, IFX: infliximab, UST: ustekinumab, VDZ: vedolizumab, BMI: body mass index, CDAI: Crohn’s Disease Activity Index, Hb: hemoglobin, and CRP: C-reactive protein.

## Data Availability

The original contributions presented in this study are included in the article. Further inquiries can be directed to the corresponding author.
